# Synthesis of New Norfloxacin–Tin Complexes to Mitigate the Effect of Ultraviolet-Visible Irradiation in Polyvinyl Chloride Films

**DOI:** 10.3390/polym14142812

**Published:** 2022-07-10

**Authors:** Marwa Fadhil, Emad Yousif, Dina S. Ahmed, Alaa Mohammed, Hassan Hashim, Ahmed Ahmed, Benson M. Kariuki, Gamal A. El-Hiti

**Affiliations:** 1 Department of Chemistry, College of Science, Al-Nahrain University, Baghdad 64021, Iraq; st.marwa.hameed@nahrainuniv.edu.iq (M.F.); emad.yousif@nahrainuniv.edu.iq (E.Y.); 2 Department of Medical Instrumentation Engineering, Al-Mansour University College, Baghdad 64201, Iraq; dina.saadi@muc.edu.iq; 3 Department of Medical Instrumentation Technical Engineering, Al-Hadi University College, Baghdad 10011, Iraq; dr.alaa.bader@huc.edu.iq; 4 Department of Physics, College of Science, Al-Nahrain University, Baghdad 64021, Iraq; hassan.hashim@nahrainuniv.edu.iq; 5 Polymer Research Unit, College of Science, Al-Mustansiriyah University, Baghdad 10052, Iraq; dr_ahmedabd@uomustansiriyah.edu.iq; 6 School of Chemistry, Cardiff University, Main Building, Park Place, Cardiff CF10 3AT, UK; kariukib@cardiff.ac.uk; 7 Department of Optometry, College of Applied Medical Sciences, King Saud University, Riyadh 11433, Saudi Arabia

**Keywords:** polyvinyl chloride films, norfloxacin–tin complexes, polymers weight loss, photodegradation, average molecular weight, surface morphology

## Abstract

Polyvinyl chloride is used in the manufacturing of a wide range of products, but it is susceptible to degradation if exposed to high temperatures and sunlight. There is therefore a need to continuously explore the design, synthesis, and application of new and improved additives to reduce the photodegradation of polyvinyl chloride in harsh environments and for outdoor applications. This research investigates the use of new norfloxacin–tin complexes as additives to inhibit the photodegradation of polyvinyl chloride to make it last longer. Reactions between norfloxacin and substituted tin chlorides, in different molar ratios and in methanol under reflux conditions, gave the corresponding organotin complexes in high yields. The chemical structures of the synthesized complexes were established, and their effect on the photodegradation of polyvinyl chloride due to ultraviolet-visible irradiation was investigated. Norfloxacin–tin complexes were added to polyvinyl chloride at very low concentrations and homogenous thin films were made. The films were irradiated for a period of up to 300 h, and the damage that occurred was assessed using infrared spectroscopy, polymeric materials weight loss, depression in molecular weight, and surface inspection. The degree of photodegradation in the polymeric materials was much less in the blends containing norfloxacin–tin complexes compared to the case where no additives were used. The use of the additives leads to a reduction in photodegradation (e.g., a reduction in the formation of short-chain polymeric fragments, weight loss, average molecular weight depletion, and roughness factor) of irradiated polyvinyl chloride. The norfloxacin–tin complexes contain aromatic moieties (aryl and heterocycle), heteroatoms (nitrogen, oxygen, and fluorine), and an acidic center (tin atom). Therefore, they act as efficient photostabilizers by absorbing the ultraviolet radiation and scavenging hydrogen chloride, peroxides, and radical species, thereby slowing the photodegradation of polyvinyl chloride.

## 1. Introduction

Plastics are potential substitutes for materials such as glass, wood, and metals in a wide range of modern applications [1,2]. Plastics can offer higher performance, they last longer, and cost less compared to other construction materials [3]. Properties such as color, density, stiffness, toughness, and transparency can be controlled during the manufacturing process of plastics. Common plastics include poly(ethylene terephthalate), polyethylene, polystyrene, polypropylene, and polyvinyl chloride (PVC) [4]. PVC additionally has excellent mechanical and chemical properties, and high resistance to environmental stress cracking. PVC is fire resistant because of its high chlorine content (ca. 57% by weight) [1]. PVC is thus used in the production of construction materials such as flooring, windows, pipes, and shutters. Other uses include upholstery, furniture, plastic cards, automobiles (e.g., sealants, fabrics, and dashboard skins), office and sports equipment, and packaging [5]. For these numerous reasons, PVC is produced on an industrial scale [6].

A downside is that PVC suffers from photochemical degradation after long-term exposure to high temperatures and sunlight [7,8]. Photodegradation of PVC alters its physical and mechanical characteristics [9]. The changes include discoloration, the appearance of cracks, and increased brittleness. This is associated with cross-linking, a decrease in average molecular weight, and weight loss, mainly due to dehydrochlorination [10,11]. PVC dehydrochlorination is promoted by defects or the presence of impurities in the polymeric chains, causing the formation of residues containing conjugated double bonds and other functional groups (carbonyl, alkene, and hydroxy) [12,13,14]. For PVC to be used for extended periods in tough outdoor environments (e.g., high temperature and humidity), its physical properties require enhancement to reduce weathering.

**Figure 1:**
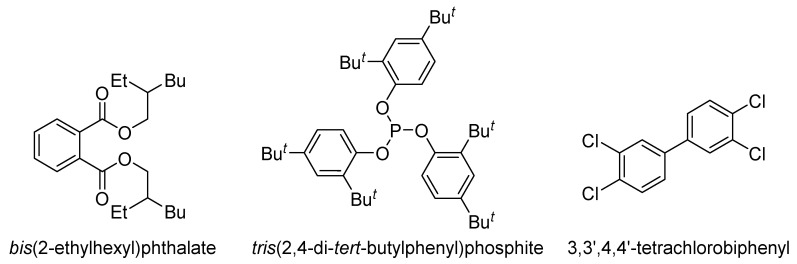
Some common PVC additives.

The performance of PVC can be improved through the incorporation of additives. An example is the addition of plasticizers to generate flexible polymeric materials [15,16]. Additives are usually aromatics used in small quantities; they are inexpensive, do not alter the color, integrate well with PVC, and are easy to produce with low waste. In addition, additives need to be chemically stable, to pose no environmental danger after use, and to be non-volatile and non-toxic. The most common industrial PVC additives include UV stabilizers, absorbers, screeners, radical scavengers, flame-retardants, and smoke suppressors [17,18,19]. Stabilizers of PVC fall into two groups. Primary stabilizers can deactivate the formation of allylic chlorides during PVC photodegradation, while secondary stabilizers act as scavengers for hydrogen chloride (HCl) and chloride radicals [19]. Some of the common PVC additives are shown in Figure 1 [20]. For example, *bis*(2-Ethylhexyl)phthalate is a non-volatile oil; it is inexpensive, PVC compatible, and has been utilized as a plasticizer. However, it has been banned as it poses a danger to health and the environment. Other PVC additives have encountered similar issues, such as *tris*(2,4-di-*tert*-butylphenyl)phosphite (an antioxidant) and 3,3′,4,4′-tetrachlorobiphenyl (a flame retardant) [21,22]. Stabilizers containing a combination of zinc and barium can be used for PVC, but co-stabilizers are needed [23,24,25].

Recently, several additives have been shown to suppress the photodegradation of PVC [26]. Examples include polybenzimidazoles [27], polyphosphates [28], Schiff bases [29,30,31,32], and pigments [33]. Various aromatic tin complexes have also been investigated as PVC additives with promising results [34,35,36,37,38,39,40,41,42,43]. In the current paper, we describe the synthesis of four novel norfloxacin–tin complexes and their effectiveness in reducing photodegradation of irradiated PVC films. Norfloxacin is commonly known for its application as an antibiotic [44]. It is a chemically stable (M.P. = 221 °C), non-toxic white solid; it is odorless, aromatic, and has a high content (34.1%) of heteroatoms (fluorine, oxygen, and nitrogen). Norfloxacin therefore has several of the properties required for an additive to be effective. Additionally, the tin atom is highly acidic (Lewis acid) and can act as a chloride radical or ion scavenger, and therefore deactivate the HCl produced due to photodegradation of PVC.

## 2. Materials and Methods

### 2.1. Common Techniques

Merck (Gillingham, UK) provided the reagents, solvents, and chemicals. PVC (*M_V_* = ca. 180,000) was sourced from Petkim Petrokimya (Istanbul, Turkey). The elemental compositions of norfloxacin–tin complexes were determined using an AA-6880 atomic absorption spectrophotometer (Shimadzu, Tokyo, Japan). The FTIR spectra were recorded on an FTIR-8300 spectrophotometer (Shimadzu, Tokyo, Japan). The NMR spectra were recorded on a Bruker BioSpin GmbH spectrometer (Bruker, Zürich, Switzerland) at 500 MHz for ^1^H and 149 MHz for the ^119^Sn measurements. A Q-Panel UV-accelerated weathering tester (Homestead, FL, USA) was used to irradiate the PVC films at 25 °C. The tester contained a stainless-steel plate which had 2 holes at the front and the back sides. Each side contained a fluorescent lamp (UV light; UV-B 365, 40 watts each). The PVC samples were fixed vertically and parallel to the UV lamps at a distance of 10 cm from the light source. The PVC samples were rotated occasionally to ensure that the films were equally irradiated from all sides. The viscosity of PVC was determined with tetrahydrofuran (THF) using an Ostwald U-Tube Viscometer (Ambala, Haryana, India). A Meiji Techno Microscope (Tokyo, Japan), an Inspect S50 microscope (FEI Company, Czechia, Czech Republic), and a Veeco instrument (Veeco Instruments Inc., Plainview, NY, USA) were used to assess the damage caused to the film surface by irradiation.

### 2.2. Synthesis of Norfloxacin–Tin Complexes ***1*** and ***2***

**Scheme 1:**
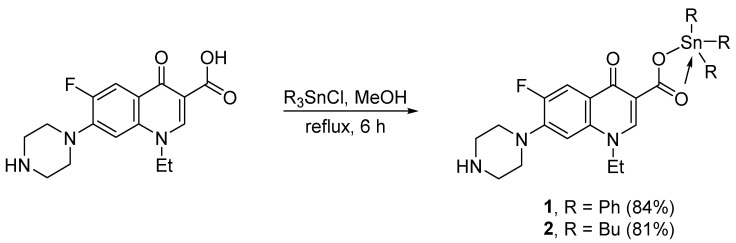
Synthesis of tin complexes **1** and **2**.

A mixture of norfloxacin (1 mmol, 319.3 mg) with either triphenyltin chloride (Ph_3_SnCl; 1 mmol, 385.5 mg) or tributyltin chloride (Bu_3_SnCl; 1 mmol, 325.5 mg) in methanol (MeOH; 20 mL) was refluxed for 6 h (Scheme 1). The mixture was allowed to cool to 25 °C and the solid was filtered, washed with MeOH (2 × 10 mL), and dried, to give either **1** (84%) as a pale yellow solid or **2** (81%) as an off-white solid (Table 1).

### 2.3. Synthesis of Norfloxacin–Tin Complexes ***3*** and ***4***

**Scheme 2:**
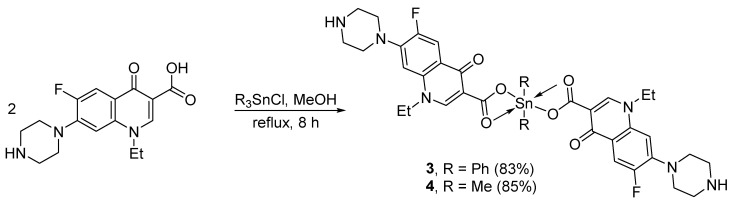
Synthesis of tin complexes **3** and **4**.

A mixture of norfloxacin (2 mmol, 638.7 mg) and diphenyltin dichloride (Ph_2_SnCl_2_; 1 mmol, 343.8 mg) or dimethyltin dichloride (Me_2_SnCl_2_; 1 mmol, 219.7 mg) in MeOH (30 mL) was refluxed for 8 h (Scheme 2). The mixture was then allowed to cool to 25 °C and the solid was filtered, washed with MeOH (2 × 10 mL), and dried, to give either **3** (83%) or **4** (85%) as off-white solids (Table 1).

### 2.4. Preparation of Formulated PVC Films

The appropriate norfloxacin–tin complex (25 mg) was added to a solution of PVC (5 g) in THF (100 mL). The mixture was stirred at 25 °C (3 h) and poured onto a glass plate perforated with 15 holes (thickness = 40 µm). The solvent was removed at 25 °C (24 h) followed by reduced pressure (8 h) in a vacuum oven at 40 °C.

### 2.5. UV-Vis Irradiation

UV light with a maximum wavelength (λ_max_) of 365 nm and an intensity of 6.2 × 10^–9^ Einstein dm^–3^ s^–1^ was used to irradiate the PVC films (0–300 h).

### 2.6. Tracking PVC Degradation

#### 2.6.1. FTIR Spectrophotometry

Photodegradation of PVC involves cleavage of the C–C bonds, and leads to the production of alcohols (hydroxyl, OH), ketones (carbonyl, C=O), and unsaturated residues (alkene, C=C) [45,46,47]. In this study, FTIR spectrophotometry was used to monitor the growth in absorption peaks corresponding to the C=O (1722 cm^−1^) and C=C (cm^−1^) bonds during the irradiation process. The growth in these peaks was compared to the intensity from the C–H bond (1328 cm^−1^), which did not change significantly during irradiation. The indices (*Is*) for the carbonyl and alkene groups (*I*_C=O_ and *I*_C=C_, respectively) were calculated using Equation (1), in which *A_s_* is the absorbance of the C=O or C=C group and *A_r_* is the absorbance of the C–H bond (reference peak) [48].

(1) $$I_{s}=\frac{A_{s}}{A_{r}}.\;$$

#### 2.6.2. Macroscopic Weight Loss

Photodegradation leads to a reduction in the weight of PVC due to bond cleavage and generation of small fragments. The percentage loss in weight can be determined from *W*_0_ (PVC weight before irradiation) and *W_t_* (weight of irradiated films) using Equation (2) [49].

(2) $$Weight\;loss\;\left(\%\right)=\frac{W_{0}-W_{t}}{W_{0}}\times 100.\;$$

#### 2.6.3. Average Molecular Weight

Photodegradation leads to smaller polymeric fragments and therefore a decrease in the average molecular weight (*M_V_*) of PVC. The intrinsic viscosity [η] can be used to determine the *M_V_* of PVC and was measured in THF for irradiated films. The *M_V_* was calculated using Equation (3) [43,50].

(3) $$\left[\eta \right]=1.63\times 10^{-2}\;M_{v}^{0.766}.\;$$

## 3. Results and Discussion

### 3.1. Synthesis of Norfloxacin–Tin Complexes ***1***–***4***

The four new norfloxacin–tin complexes were obtained in high yields. The reaction of norfloxacin and triphenyltin or tributyltin chloride in a 1:1 ratio gave the corresponding complexes **1** or **2**, respectively (Scheme 1). The reaction of two mole equivalents of norfloxacin and diphenyltin or dimethyltin chloride gave the corresponding complexes **3** or **4** (Scheme 2). Some physical properties of norfloxacin–tin complexes **1**–**4** are shown in Table 1. The FTIR spectra of complexes **1**–**4** revealed the disappearance of the –OH absorption band which appears at 3367 cm^−1^ for norfloxacin. The bonds between the tin and carbon (Sn–C) and oxygen (Sn–O) atoms gave rise to absorption bands in the regions of 532–559 and 440–472 cm^−1^, respectively. The spectra also showed strong absorption bands in the 1383–1466 and 1614–1651 cm^−1^ regions, due to symmetrical and asymmetrical vibrations in the carboxylate unit (COO^–^), respectively (Table 2). The difference between the frequencies of the asymmetrical and symmetrical vibrations (∆ν) was 171–231 cm^−1^. The ∆ν value indicates a bi-dentate mode of coordination for the carboxylate group [51]. Thus, the norfloxacin is coordinated to the tin atom via the two oxygen atoms in the carboxylate group.

The ^1^H NMR spectra of complexes **1**–**4** revealed the absence of the carboxylate proton of norfloxacin. The spectra showed all the expected protons from both tin substituents and norfloxacin (Table 3). The ethyl protons appeared as triplet and quartet signals in the high field region. Two quinolinyl protons appeared as doublets (*J* = ca. 16 and 4 Hz) due to coupling with the fluorine atom. The ^149^Sn NMR spectra indicated that coordination had taken place between the tin atom and norfloxacin, and showed singlet signals in the region between –152.1 and –399.8 ppm (Table 3).

### 3.2. Characterization of the Ageing

#### 3.2.1. FTIR Spectrophotometry

**Figure 2:**
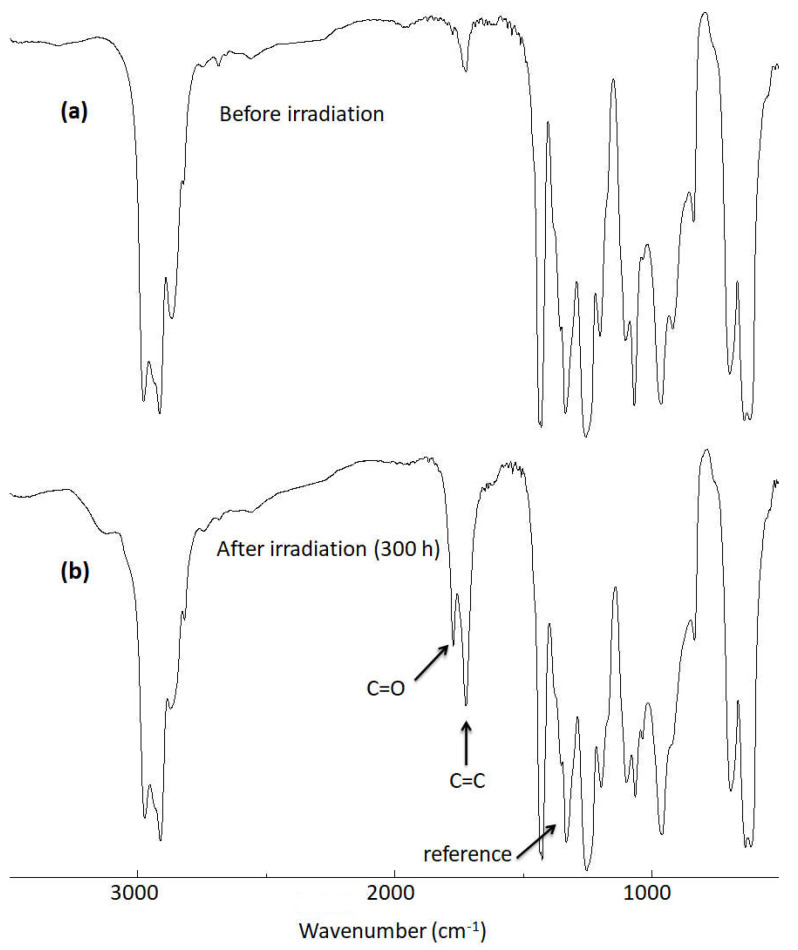
FTIR spectra of (**a**): blank PVC film before irradiation and (**b**): the PVC film after irradiation (300 h).

Irradiation of PVC in an oxygen-rich environment can cause photooxidative degradation. In the process, smaller polymeric fragments containing C=O (ketones; 1722 cm^−^^1^) and C=C (unsaturated residues; 1602 cm^−^^1^) groups can be formed [45,46,47]. The rate of PVC photooxidation was monitored using FTIR spectroscopy. The intensity of absorption bands due to both the C=O and C=C groups were determined and compared with that of a reference bond (the absorption band of C–H; 1328 cm^−^^1^). The intensity of the C–H absorption band remained relatively unchanged during the irradiation process. Figure 2 shows the increase in the intensities of both C=O and C=C absorption bands for the blank PVC film after irradiation.

**Figure 3:**
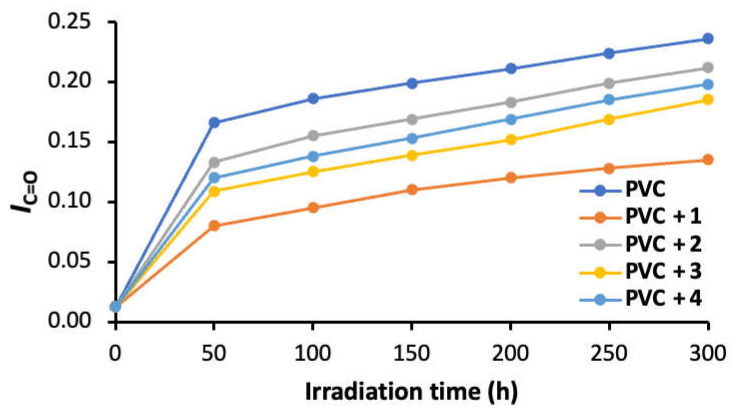
A plot showing the evolution of the *I*_C=O_ for PVC films as a function of irradiation time.

**Figure 4:**
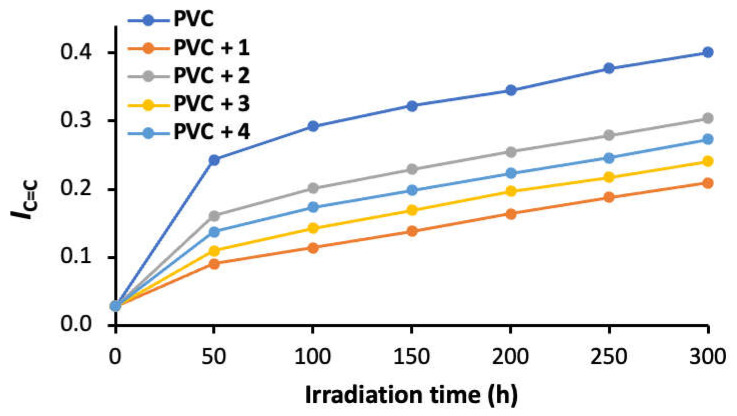
A plot showing the evolution of the *I*_C=C_ for PVC films as a function of irradiation time.

Equation (1) was used to calculate the *I*_C=O_ and *I*_C=C_ for the PVC films after different irradiation times, ranging from 50 to 300 h. Figure 3 and Figure 4 show a higher level of both *I*_C=O_ and *I*_C=C_ in the absence of norfloxacin–tin complexes **1**–**4**. The use of additives, particularly tin complex **1**, led to significant lowering in both *I*_C=O_ and *I*_C=C_. The efficiency of **1**–**4** as PVC additives follows the order of aromaticity content within the complexes (i.e., **1** > **3** > **4** > **2**). This is illustrated by the *I*_C=O_ values of 0.24 (blank PVC), 0.14 (PVC + **1**), 0.21 (PVC + **2**), 0.19 (PVC + **3**), and 0.20 (PVC + **4**) after 300 h of irradiation, compared with 0.01 for the non-irradiated films. After 300 h of irradiation, the *I*_C=C_ values were 0.40 (blank PVC), 0.21 (PVC + **1**), 0.30 (PVC + **2**), 0.24 (PVC + **3**), and 0.27 (PVC + **4**), compared with 0.03 for the non-irradiated films. Additives **1** and **3** contain aromatic rings (phenyl groups), while **2** and **4** contain aliphatic substituents. This explains why additives **1** and **3** performed better as photostabilizers compared with **2** and **4**. Also, additive **4,** which contained the non-bulky methyl groups, was more efficient than additive **2,** which had the bulky butyl groups. The steric hindrance tended to reduce the effect of the tin atom as a scavenger for HCl.

#### 3.2.2. Macroscopic Weight Loss

**Figure 5:**
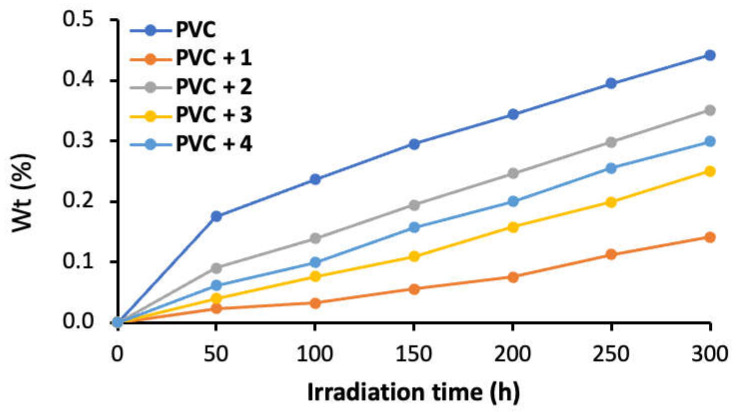
A plot showing the percentage weight loss (%) for PVC films as a function of irradiation time.

Dehydrochlorination of PVC takes place mainly through cross-linking and chain scission. The elimination of volatile products (e.g., HCl) causes the formation of unsaturated residues, accompanied by PVC weight loss [45,49]. In the investigation, the PVC films were irradiated and the percentage weight loss (%) was determined using Equation (2). In comparison to the blank film, weight loss from irradiation was lower when the norfloxacin–tin complexes **1**–**4** were blended (Figure 5). Complex **1** resulted in less weight loss than the other additives. Thus, for example, the percentage weight loss after 100 h of irradiation was 0.24 (blank PVC), 0.03 (PVC + **1**), 0.14 (PVC + **2**), 0.08 (PVC + **3**), and 0.10 (PVC + **4**). The values after 300 h of irradiation were 0.44 (blank PVC), 0.14 (PVC + **1**), 0.35 (PVC + **2**), 0.25 (PVC + **3**), and 0.30 (PVC + **4**).

#### 3.2.3. Average Molecular Weight (M_V_)

**Figure 6:**
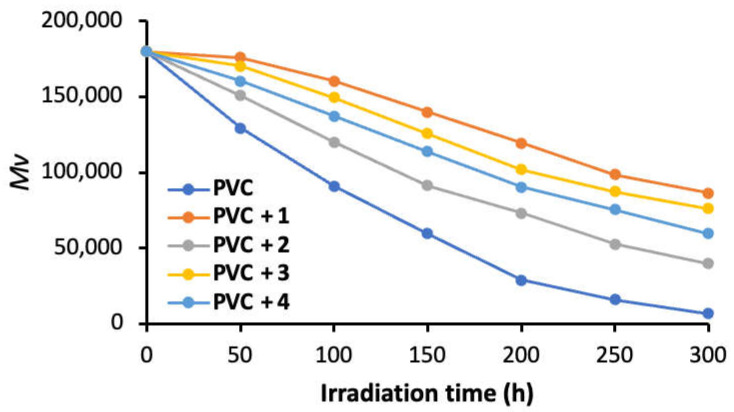
A plot showing *Mv* for PVC films as a function of irradiation time.

Irradiation of PVC leads to a decrease in *M_V_*. The *M_V_* are directly proportional to the [η] of solutions of the irradiated films [50]. For determination, the PVC films were irradiated, dissolved in THF, and the [η] was measured using a viscometer. The *M_V_* were calculated after different irradiation times using Equation (3). Figure 6 shows that the *M_V_* depression was fastest and sharpest in the absence of any additives. The value of the blank PVC film decreased from ca. 180,000 to ca. 90,844 (50% loss in *M_V_*) after 100 h of irradiation and to only 6799 (96% loss in *M_V_*) at the end of the irradiation process. The use of norfloxacin–tin complexes inhibited the decrease in the *M_V_* significantly. So, for example, the use of complex **1** reduced the decrease to ca. 160,453 (11% loss in *M_V_*) and to 86,301 (52% loss in *M_V_*) after 100 and 300 h of irradiation, respectively. Thus, the norfloxacin–tin complexes clearly inhibit the deterioration of *M_V_* and protect the PVC films.

#### 3.2.4. Surface Analysis of PVC Films

Optical microscopy, scanning electron microscope (SEM), and atomic force microscopy (AFM) images can detect surface irregularities in PVC films due to irradiation [52,53,54,55,56]. The microscopic images of the irradiated PVC films showed that the additives played a major role in stabilizing the polymer against irradiation. The surfaces of the irradiated PVC films containing norfloxacin–tin complexes showed less damage compared to the blank film (Figures S1–S6). The least surface damage was seen in the film containing additive **1** which is the complex with the most aromatic content. The AFM images (Figures S5 and S6) showed that surface of the PVC film containing no additives was rough and irregular after irradiation. The surfaces of the PVC blends containing additives, and in particular those containing **1** and **2**, were less rough after irradiation compared with the blank film. For the pure PVC film, the roughness factor (*R*q) was 468, whereas the values were 36.3 for PVC + **1**, 62.8 PVC + **2**, 41.2 for PVC +**3**, and 46.2 for PVC + **4**. Complex **1** led to an improvement in the *R*q of PVC by 12.9-fold, which was the largest for the additives. The reduction in the *R*q using **1**–**4** was higher than those obtained with other additives, such as tin complexes of naproxen (*R*q = 5.2) [34], carvedilol (*R*q = 6.4) [35], furosemide (*R*q = 6.6) [36], captopril (*R*q = 7.0) [37], valsartan (*R*q = 7.4) [38], telmisartan (*R*q = 9.4) [39], and trimethoprim (*R*q = 6.6) [11.3]. However, tin complexes of ciprofloxacin (*R*q = 16.6) [41], 4-(benzylideneamino)benzenesulfonamide (*R*q = 18.4) [42], and of 4-methoxybenzoic acid (*R*q = 16.6) [43] led to greater reduction in the *R*q of the irradiated PVC films. These additives have higher content of aromatic moieties and heteroatoms compared with those investigated in the current study.

### 3.3. Mechanism of PVC Photostabilization by Additives

**Scheme 3:**
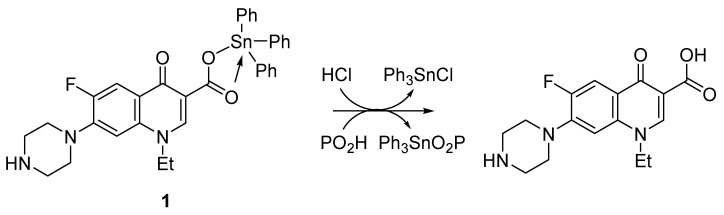
Complex **1** acts as a hydrogen chloride and peroxide scavenger.

PVC photostabilization was significantly greater in the presence of norfloxacin–tin complexes **1**–**4** and, in particular, the additives with the highest aromatic content (i.e., complexes **1** and **3**). The reason is that coordination between the electronegative and electropositive centers in complexes and PVC can lead to stabilization of the polymeric chains against degradation. The tin atom is acidic (secondary stabilizer) and tends to deactivate HCl (i.e., it is Cl radical or ion scavenger) eliminated from the PVC films during irradiation. Therefore, substantial stabilization has been achieved in the presence of additives **1**–**4** [27]. As an example, tin complex **1** deactivates the HCl generated on PVC photodegradation to produce triphenyltin chloride (Scheme 3). In environments containing active oxygenated species (e.g., hydrogen peroxide), PVC suffers from photooxidation [11]. The tin additives containing norfloxacin (e.g., complex **1**) decompose hydroperoxides (Scheme 3) and therefore protect the PVC films against degradation.

**Scheme 4:**
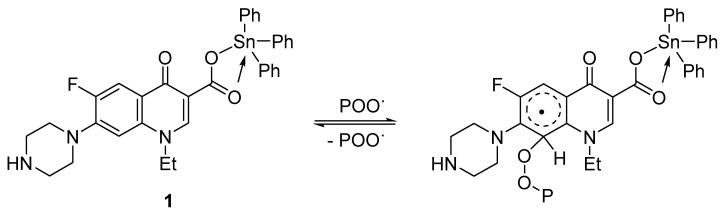
Complex **1** acts as a peroxide radical scavenger.

Various photooxidative products can be produced when PVC reacts with peroxide radicals (POO^•^) [57]. Norfloxacin–tin complexes (e.g., complex **1**) scavenge the radicals and form stable intermediates via resonance (Scheme 4). Therefore, complexes **1**–**4** tend to reduce the harmful effects of peroxide radicals on the PVC.

## 4. Conclusions

Four new tin–norfloxacin complexes were synthesized using simple procedures. The use of tin complexes at a low concentration has been proven to be effective in inhibiting photodegradation of irradiated polyvinyl chloride films. The effectiveness of the synthesized complexes as photostabilizers was higher compared with many of the reported organometallics. The complexes containing a high aromatic content (phenyl groups) acted as better photostabilizers to polyvinyl chloride compared with those containing just aliphatic substituents (methyl and butyl groups) The tin–norfloxacin complexes protect polyvinyl chloride by acting as ultraviolet radiation absorbers and hydrogen chloride, peroxide, and radical scavengers. For potential application, the possible leakage of tin to the surrounding environments will need to be assessed. The impact of photodegradation necessitates continued research in the design and synthesis of new non-toxic additives, and their effectiveness as photostabilizers in the suppression of the photodegradation of plastics.

## Figures and Tables

**Table 1:** Synthesis and some physical properties data for **1**–**4**.

Complex	Color	R	M.P. (°C)	Yield (%)	Calculated (Found; %)			
					C	H	N	Sn
**1**	Pale yellow	Ph	245–246	84	61.98 (62.10)	4.81 (4.83)	6.20 (6.29)	17.74 (17.76)
**2**	Off-white	Bu	225–226	81	55.25 (55.28)	7.25 (7.29)	6.89 (6.91)	19.48 (19.51)
**3**	Off-white	Ph	235–236	83	58.78 (58.60)	4.85 (4.88)	9.23 (9.24)	13.01 (13.05)
**4**	Off-white	Me	227–228	85	51.99 (52.02)	5.13 (5.16)	10.70 (10.71)	15.11 (15.12)

**Table 2:** The FTIR absorption bands of complexes **1–4**.

Complex.	FTIR, Frequency (*ν*, cm^−1^)					
	C=O	Carboxylate Group (COO^−^)			Sn–C	Sn–O
		asym	sym	Δν (asym − sym)		
**1**	1680	1622	1426	196	540	440
**2**	1685	1614	1383	231	559	472
**3**	1682	1651	1466	185	553	461
**4**	1682	1620	1449	171	532	463

**Table 3:** ^1^H and ^119^Sn NMR spectral data for **1–4**.

Complex	1H NMR	^119^Sn NMR
**1**	8.46 (s, 1H, quinolinyl), 7.96–7.32 (m, 17H, quinolinyl and 3Ph), 3.83 (s, exch., 1H, NH), 3.75 (q, *J* = 7.6 Hz, 2H, CH_2_), 3.43 (s, 4H, piperazinyl), 2.75 (s, 4H, piperazinyl), 1.34 (t, *J* = 7.6 Hz, 3H, Me)	–175.1
**2**	8.68 (s, 1H, quinolinyl), 7.93 (d, *J* = 16.0 Hz, 1H, quinolinyl), 7.61 (d, *J* = 4.0 Hz, 1H, quinolinyl), 3.83 (s, exch., 1H, NH), 3.53 (q, *J* = 7.7 Hz, 2H, CH_2_), 3.34 (s, 4H, piperazinyl), 2.46 (s, 4H, piperazinyl), 1.65–0.92 (m, 30H, Me and 3Bu)	–152.1
**3**	8.68 (s, 2H, quinolinyl), 7.96 (d, *J* = 15.0 Hz, 2H, quinolinyl), 7.61 (d, *J* = 4.0 Hz, 2H, quinolinyl), 7.39–7.16 (m, 20H, 4Ph), 3.48 (q, *J* = 7.5 Hz, 4H, 2CH_2_), 3.89 (s, exch., 2H, 2NH), 3.74 (s, 8H, piperazinyl), 2.80 (s, 8H, piperazinyl), 1.35 (q, *J* = 7.5 Hz, 6H, 2Me)	–399.8
**4**	9.10 (s, 2H, quinolinyl), 7.53 (d, *J* = 14.0 Hz, 2H, quinolinyl), 7.39 (d, *J* = 4.0 Hz, 2H, quinolinyl), 4.97 (s, exch., 2H, 2NH), 3.68 (q, *J* = 7.7 Hz, 4H, 2CH_2_), 3.43 (s, 8H, piperazinyl), 2.80 (s, 8H, piperazinyl), 1.41 (s, 6H, 2Me), 0.89 (t, *J* = 7.7 Hz, 6H, 2Me)	–230.2

## Data Availability

Data are contained within the article.

## Supplementary Materials

The following supporting information can be downloaded at: https://www.mdpi.com/article/10.3390/polym14142812/s1, Figure S1: microscopy images of unblended PVC film (a): before irradiation and (b): after irradiation. Figure S2: microscopy images of irradiated PVC films containing (a): complex **1**, (b): complex **2**, (c): complex **3**, and (d): complex **4**. Figure S3: SEM images of PVC film (a): before irradiation and (b): after irradiation. Figure S4: SEM images of irradiated PVC films containing (a): complex **1**, (b): complex **2**, (c): complex **3**, and (d): complex **4**. Figure S5: two- and three-dimensional AFM images of the blank PVC film (a): before irradiation and (b): after irradiation. Figure S6: two- and three-dimensional AFM images of irradiated PVC films containing (a): complex **1**, (b): complex **2**, (c): complex **3**, and (d): complex **4**.
