# Supplementary material for: Synthesis of New Norfloxacin–Tin Complexes to Mitigate the Effect of Ultraviolet-Visible Irradiation in Polyvinyl Chloride Films
Source: Polymers (Basel). 2022 Jul 10;14(14):2812. doi: 10.3390/polym14142812 (PMC9324004; doi:10.3390/polym14142812)
Supplement: Supplementary file 1 [file polymers-14-02812-s001.zip › polymers-1784690-supplementary.pdf]

polymers

Supplementary Materials

Synthesis of New Norfloxacin-Tin Complexes to Mitigate the Effect of Ultraviolet-Visible Irradiation in Polyvinyl Chloride Films

Marwa Fadhil [1], Emad Yousif [1], Dina S. Ahmed [2], Alaa Mohammed [3], Hassan Hashim [4], Ahmed Ahmed [5], Benson M. Kariuki [6] and Gamal A. El-Hiti [7,*]

[1] Department of Chemistry, College of Science, Al-Nahrain University, Baghdad 64021, Iraq; st.marwa.hameed@ced.nahrainuniv.edu.iq (M.F.); emad.yousif@ced.nahrainuniv.edu.iq (E.Y.)
[2] Department of Medical Instrumentation Engineering, Al-Mansour University College, Baghdad 64021, Iraq; dina.saadi@muc.edu.iq
[3] Department of Medical Instrumentation Technical Engineering, Al-Hadi University College, Baghdad 64021, Iraq; dr.alaa.bader@huc.edu.iq
[4] Department of Physics, College of Science, Al-Nahrain University, Baghdad 64021, Iraq; hassan.hashim@nahrainuniv.edu.iq
[5] Polymer Research Unit, College of Science, Al-Mustansiriyah University, Baghdad 10052, Iraq; dr_ahmedabd@uomustansiriyah.edu.iq
[6] School of Chemistry, Cardiff University, Main Building, Park Place, Cardiff CF10 3AT, UK; kariukib@cardiff.ac.uk
[7] Department of Optometry, College of Applied Medical Sciences, King Saud University, Riyadh 11433, Saudi Arabia
* Correspondence: gelhiti@ksu.edu.sa; Tel.: +966-11469-3778; Fax: +966-11469-3536

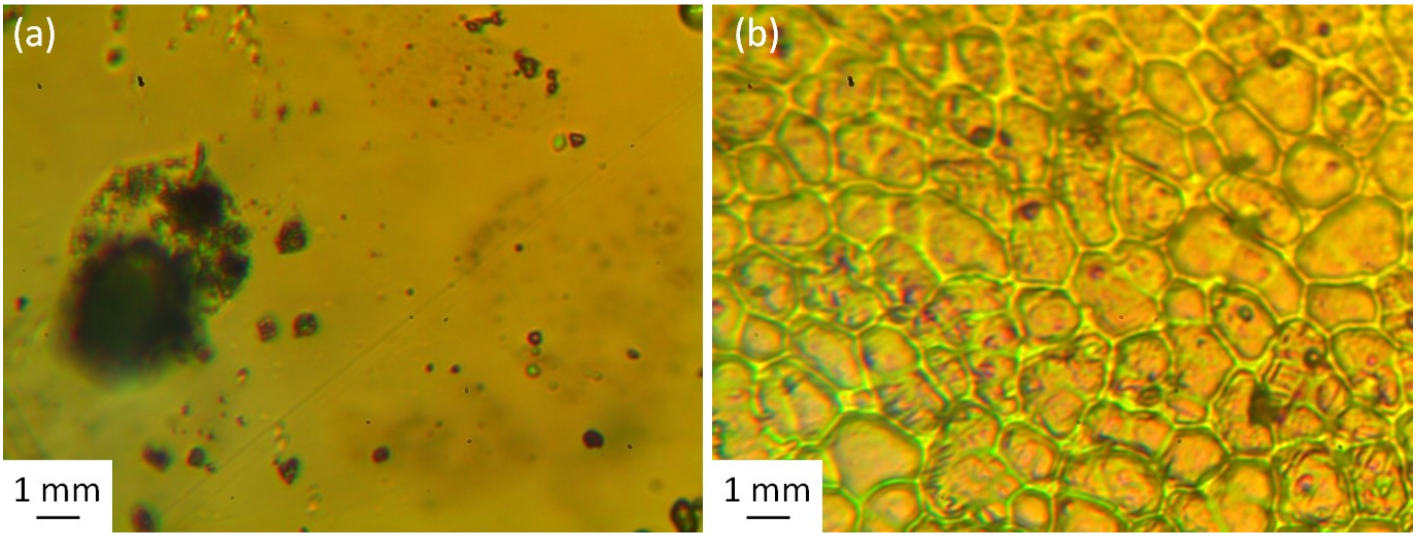

Figure S1. Microscopy images (400× magnification) of unblended PVC film (a): before irradiation and (b): after irradiation.

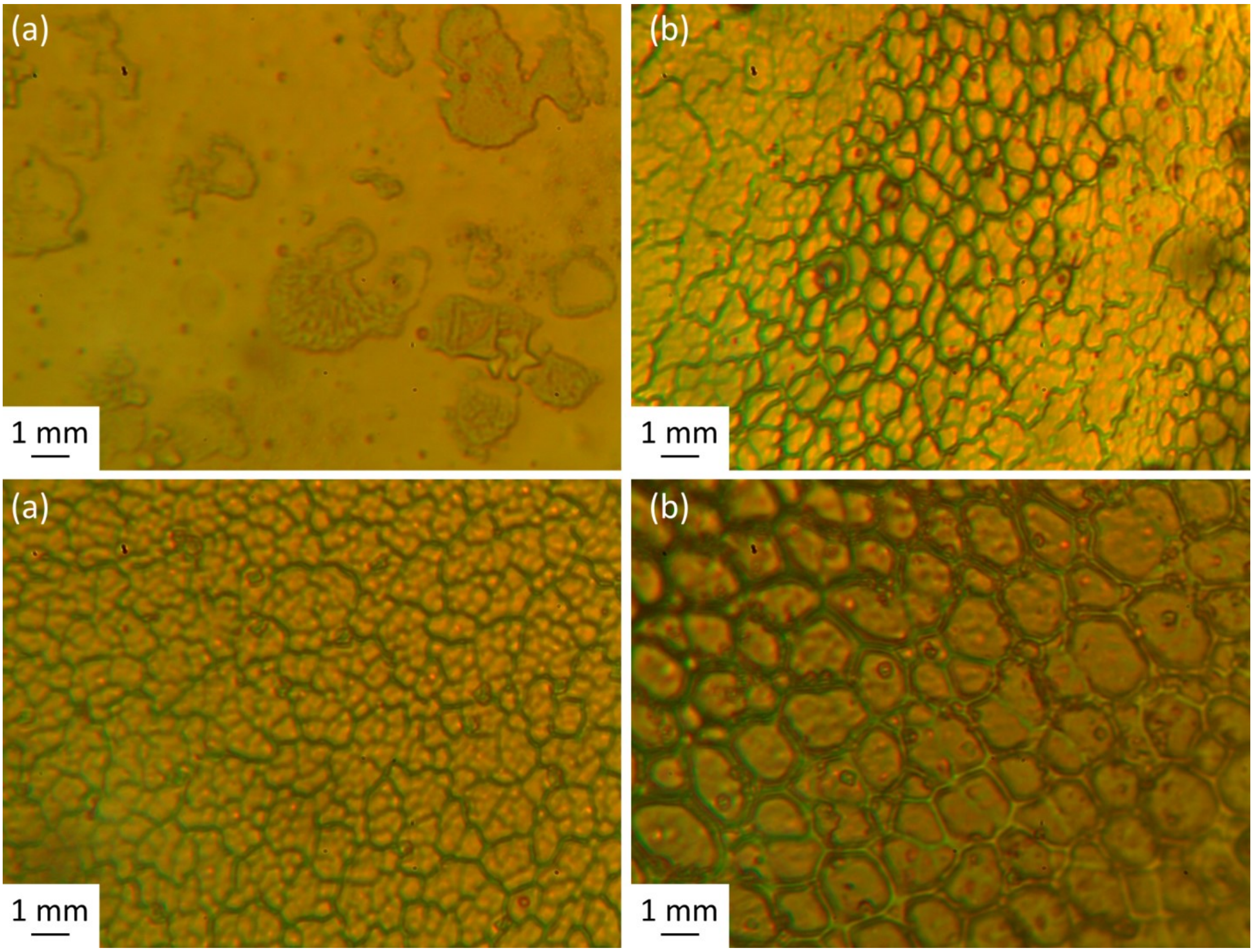

Figure S2. Microscopy images (400× magnification) of irradiated PVC films containing (a) complex **1**, (b): complex **2**, (c): complex **3**, and (d): complex **4**.

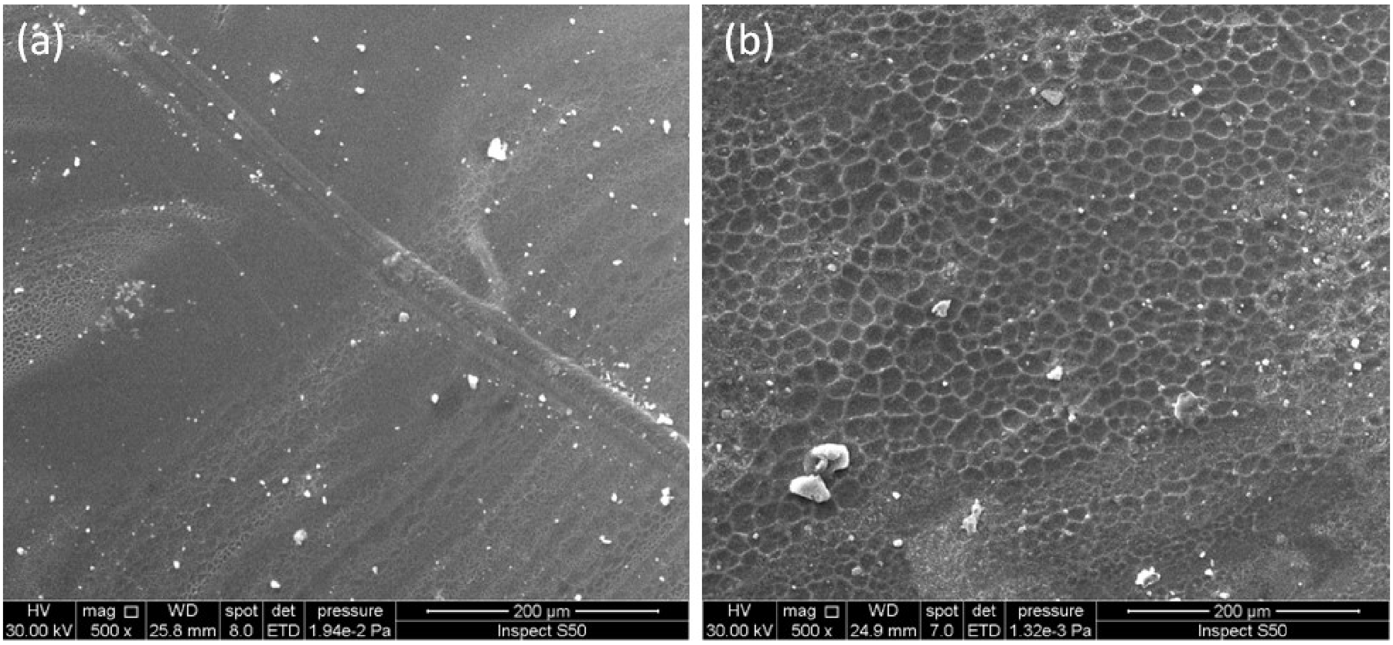

Figure S3. SEM images of PVC film (a): before irradiation and (b): after irradiation.

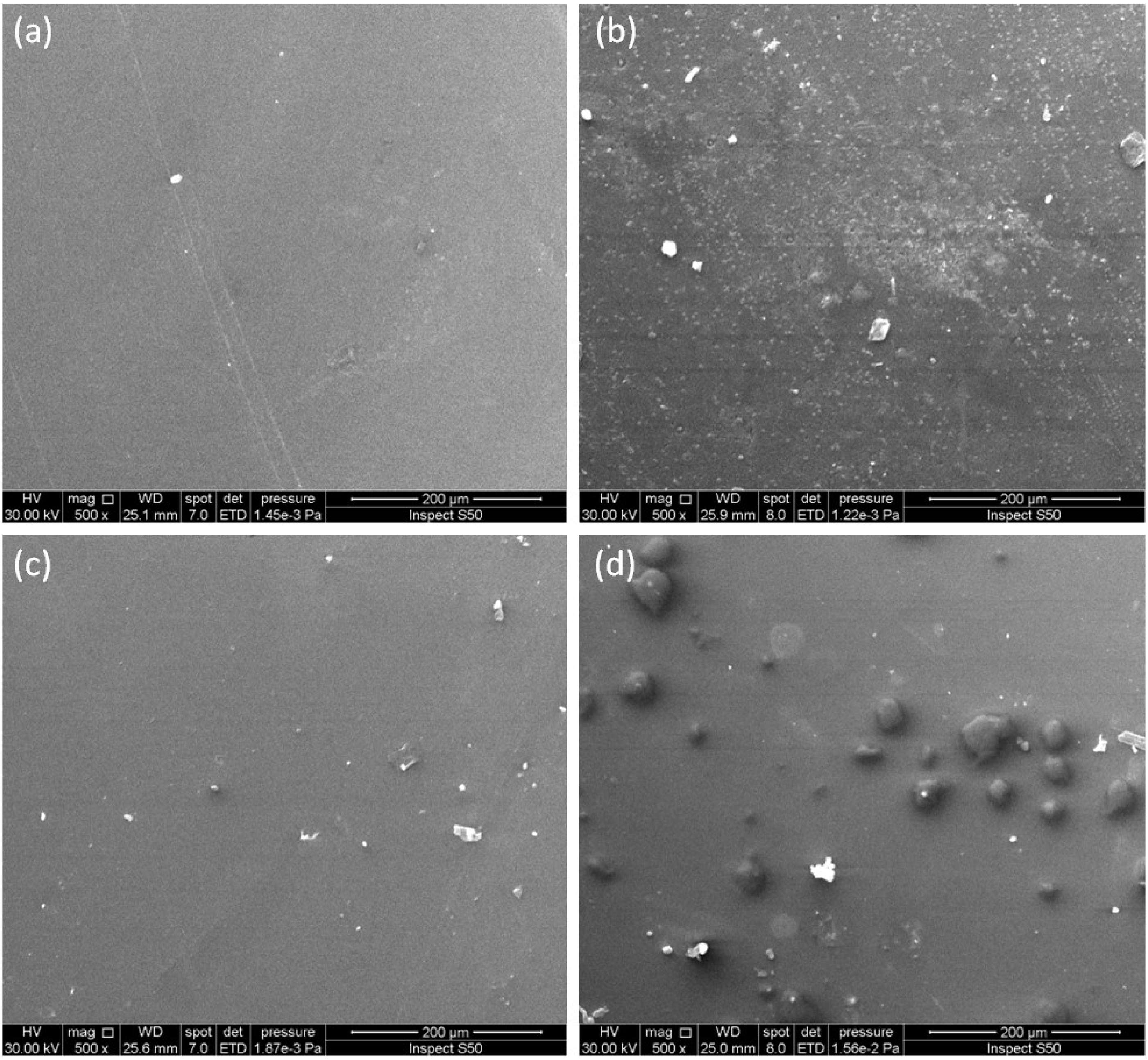

Figure S4. SEM images of irradiated PVC films containing (a) complex **1**, (b): complex **2**, (c): complex **3**, and (d): complex **4**.

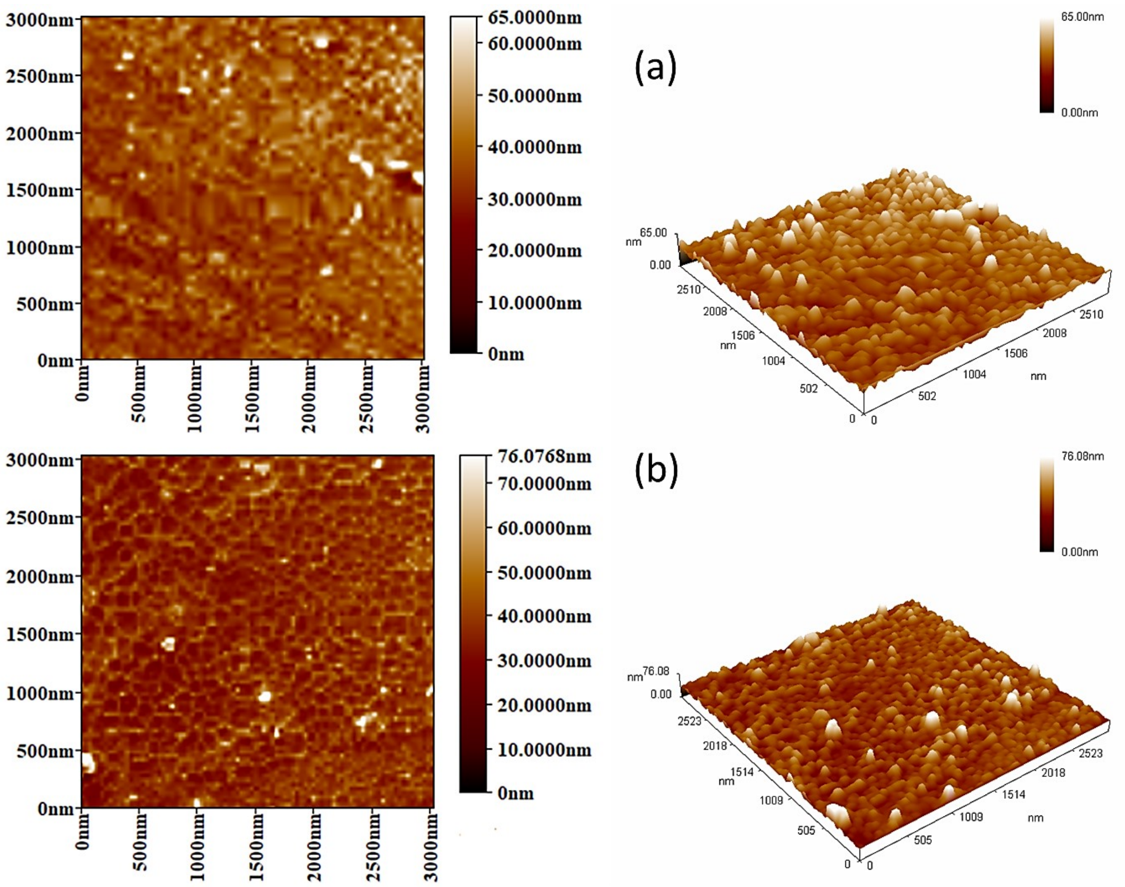

Figure S5. Two- and three-dimensional AFM images of the blank PVC film (a): before irradiation and (b): after irradiation.

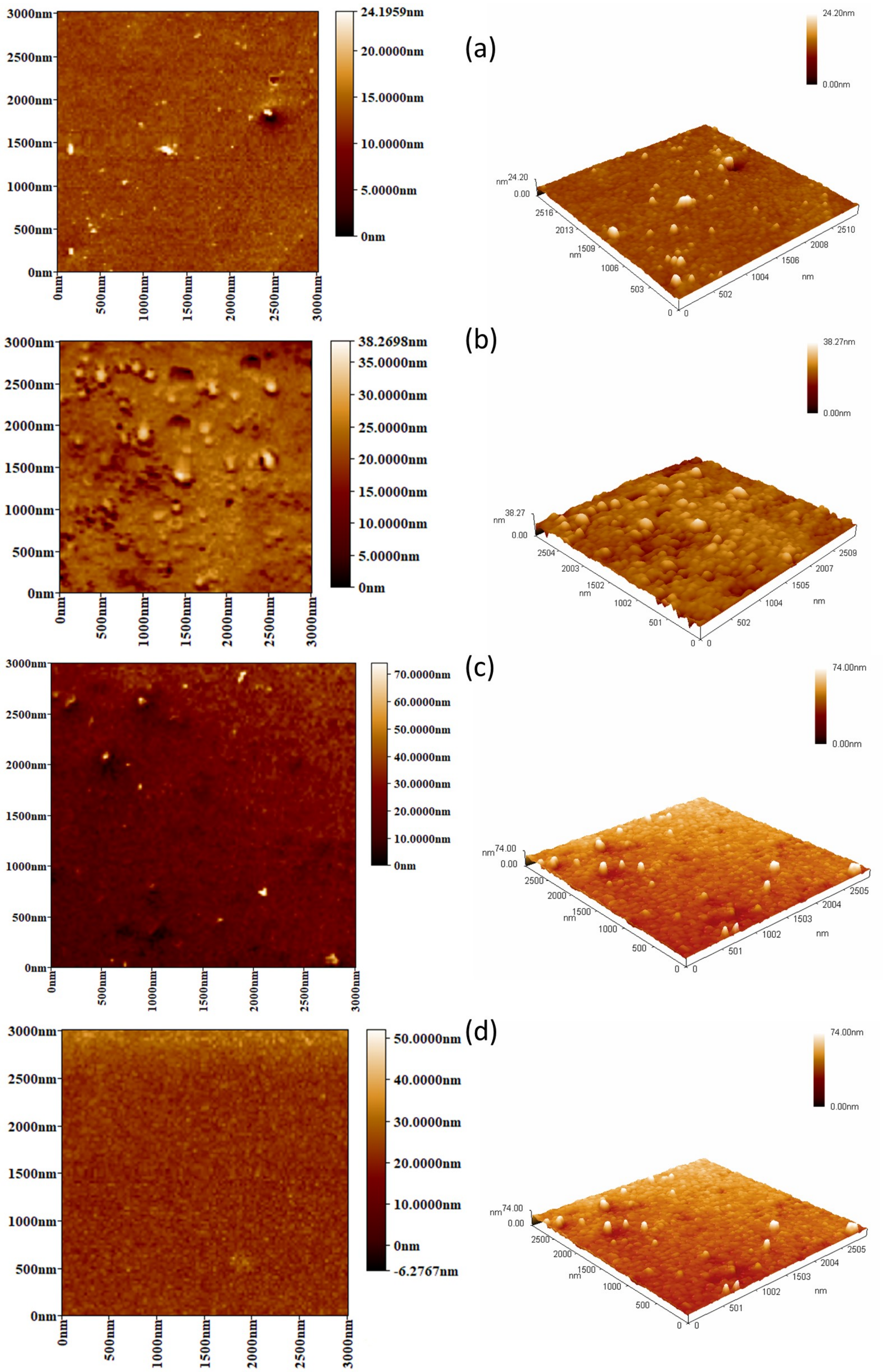

Figure S6. Two- and three-dimensional AFM images of irradiated PVC films containing (a) complex **1**, (b): complex **2**, (c): complex **3**, and (d): complex **4**.
